# Qualitative study of the clinician–parent interface in discussing prognosis following MRI and US imaging of preterm infants in the UK

**DOI:** 10.1136/bmjopen-2016-011472

**Published:** 2016-09-26

**Authors:** M E Harvey, M E Redshaw

**Affiliations:** 1 Department of Perinatal Imaging and Health, Division of Imaging and Biomedical Engineering, King's College, London, UK; 2 National Perinatal Epidemiology Unit, University of Oxford, Oxford, UK; 3 Faculty of Health, Education and Life Sciences, Birmingham City University, Birmingham, UK

**Keywords:** communication, MRI, preterm, parents, ultrasound scan

## Abstract

**Objective:**

To explore communication and interaction between parents and clinicians following neonatal ultrasound (US) and MRI of the brain of babies born preterm.

**Setting:**

This qualitative study was undertaken as part of a larger UK study of neonatal brain imaging. 511 infants were cared for in 14 London neonatal units with MR and cerebral US imaging in a specialist centre.

**Participants:**

Parents with infants born at <33 weeks gestation were randomised to receive prognostic information based on either MRI or US findings on their infants at term-corrected age.

**Methods:**

Discussions between parents and clinicians about the MRI or US result were audio recorded. Parents were told about the findings and their baby's predicted outcome. A topic guide ensured essential aspects were covered. Recordings were fully transcribed. Discussion of the scan results, the content and style of the interaction and parental response were analysed qualitatively in 36 recordings using NVivo V.10.

**Outcomes:**

Key themes and subthemes were identified in the clinician–parent discussions.

**Results:**

The overarching theme of ‘the communication interface’ was identified with three key themes: ‘giving information’, ‘managing the conversation’ and ‘getting it right’ and further subthemes. A range of approaches were used to facilitate parental understanding and engagement. There were differences in the exchanges when information about an abnormal scan was given. The overall structure of the discussions was largely similar, though the language used varied. In all of the discussions, the clinicians talked more than the parents.

**Conclusions:**

The discussions represent a difficult situation in which the challenge is to give and receive complex prognostic information in the context of considerable uncertainty. The study highlights the importance of being able to re-visit specific issues and any potential areas of misunderstanding, of making time to talk to parents appreciating their perspective and level of knowledge.

**Trial registration number:**

EudraCT 2009-013888-19; Pre-results.

**Strengths and limitations of this study**

- Audio-recording and analysis of clinician–parent discussions is rare.
- The qualitative analysis of the interaction with parents provides insights with the potential to inform and change practice.
- A large proportion of the parents participating in the main study were willing to participate in this qualitative and well represented the diverse population served by the participating study sites.
- Data collection took place in the context of a trial and might not necessarily reflect routine clinical interactions.
- Video-recording would have allowed non-verbal cues to be documented, but would have had the potential to be more intrusive.

## Background

This qualitative study was undertaken as part of a larger programme of research on neonatal brain imaging in which the main element was a trial. Following an MRI and ultrasound (US) scans at term, babies born before 33 weeks gestation were randomised and parents received prognostic information about the baby based on either the MRI or US result (ePrime study). The hypothesis of the larger study related to a reduction in parental anxiety following provision of more detailed information based on MRI. The purpose of this qualitative study was to explore the communication and interaction that occurred during the provision of prognostic information based on the scans.

Effective communication between healthcare professionals and parents is considered a fundamental aspect of family-centred care.^1^ ^2^ Qualitative research has focused on broad aspects of clinician–parent communication,^3–6^ and a systematic review has explored possible interventions.^7^ The aim of the review was to identify and map out effective interventions in communicating and providing information to parents of preterm infants. The evidence suggested that communication interventions by which parents are prepared for care in the neonatal unit, informed and supported throughout the infant's stay and after discharge are of potential benefit, though the study quality reported was mixed. Very little research has focused specifically on communication between clinicians and parents about brain imaging; however, this was an aspect of a small-scale qualitative study of parents' experiences of information-giving in the neonatal unit^8^ which showed that most felt they initially were passive recipients of information, accessing specific information such as test results with difficulty. It seemed that concerns about long-term developmental outcome continued and the emotional impact of having a preterm baby negatively affected parents' ability to retain information. Accounts of one couple's experience of information-giving after an MRI at term of their preterm baby^9^ and the responses of the clinicians involved^10^ suggest that MRI scanning results in this situation could be less than helpful to parents. These small-scale studies and accounts have identified some of the challenges and difficulties encountered by clinicians and parents during the provision of information, particularly when the information is complex and has far-reaching significance for families.^6^ ^8^ ^10^

Audio-recorded discussions between parents and paediatricians have been used in a small number of studies of parent–clinician communication.^11–15^ In some of these studies, the main aim was to facilitate parental understanding and recall, rather being a way of investigating the communication process.^11–13^ In one study, audio-recordings of clinician–parent communication about the child's possible participation in a clinical trial were analysed.^14^ During these discussions, the clinicians generally used closed questions and parents said very little, asking few questions. Another study involved seven families of children with dysmorphic features during which their discussions with clinicians were recorded.^15^ Analysis revealed the impact of discussion about more difficult issues such as the child's appearance and the longer term. At these points, the discussions were more disjointed with limited parental involvement. No published studies have been identified using audio-recording to specifically investigate how diagnostic information is discussed in talking with parents of preterm infants. While an earlier analysis focused on clinician strategies,^16^ the aim of the present study was to explore the communication process and content of the discussions between parents and clinicians about neonatal brain imaging.

## Methods

Babies were recruited to the larger study while being cared for in one of 14 neonatal units in the London area (EudraCT reference: 2009-011602-42, Clinicaltrials.gov: NCT01049594. ePrime: Evaluation of MRI to Predict Neurodevelopmental Impairment in Preterm Infants).

When consenting to the larger study, parents were asked if they would also be willing for the discussion about the imaging result to be recorded and most parents or ‘family units’ agreed (80% of those participating in the larger study, 350 out of 434).

After discharge home, all babies attended a hospital with neonatal imaging facilities for MRI and US scans when they reached term equivalent. Written informed consent was obtained at the recruitment site. Parental consent was affirmed at the scanning appointment, and randomisation took place after both scans had taken place. Parents were given either the MRI or US scan result by one of three clinicians (two consultants and a senior research fellow), all of whom were informed by the published evidence and experienced in discussing imaging of perinatal brain injury and prognosis with parents. No individual assessments were made of clinician knowledge.

This took place in a quiet, private room. The report and images arising from the randomised result (MRI or US) were only made available to the clinicians after the imaging and just prior to the discussion with parents. The clinicians giving the diagnostic information were based at the main study site and so local variation at other sites in use of MRI and cerebral ultrasound (CUS) would not have affected how the information was given.

The purpose of the discussion was to give parents the scan findings and to provide information about the baby's possible long-term outcomes. A topic guide/script ensured that essential information was given in a generally agreed order (box 1). Images from the scan were also used to aid the communication process. Copies of the randomised image (MRI or US) were given to parents on the day of the scan, all parents were sent a letter summarising the information given and if they had participated in this study, a copy of the audio recording was offered. A total of 60 recordings were made of consecutive parent–clinician discussions over three specific time periods: during the early, middle and late phases of data collection. The clinicians did not select the discussions to be recorded. These time points were chosen to capture any differences that might occur over the course of the study.

Box 1 **Topic guide used to facilitate the provision of essential information**

- Randomisation and how the results will be given.
- What parents have previously been told about scan results and the baby's prognosis.
- An overview of the MRI or ultrasound result.
- More detailed information about the scan using the images to explain the findings.
- General long-term risks of problems for babies born preterm with specific reference to cerebral palsy and learning difficulties.
- Prognosis for the baby based on the scan result, with reference to risk of cerebral palsy and learning difficulties.

All of the audio-recordings were transcribed, and based on the first 24 of these and the literature from other healthcare settings on clinician–patient interaction,^17–21^ a framework was developed.^16^ For the present study of the style and pattern of communication between clinicians and parents in the context of giving diagnostic and prognostic information, 36 recordings were selected without reference to content but which included equal numbers of families receiving information based on the two different types of scanning information, families where cerebral abnormalities had been identified in their infants and those from a range of backgrounds. Reflecting the diversity of the participants from across the 3 years of the study, 12 recordings for each of three clinicians were analysed thematically. The focus was the content and interaction between the participants. NVivo V.10 facilitated this process with both researchers reviewing the transcripts separately in an iterative manner using constant comparison.^22^ After initial coding and review, the researchers met to compare interpretation, agree on coding and the key themes and subthemes identified. Approvals for the larger study programme of work, of which this was part, were obtained from the Hammersmith, Queen Charlotte's and Chelsea Research Ethics Committee (Number: 09/H0707/87).

## Results

The recordings analysed concerned the outcomes of 43 preterm babies (30 singletons, 6 multiples) whose families were recruited from 11 sites, 2 of which were tertiary centres (table 1). For nearly half the recordings, both parents were present and for the remainder, took place with one parent, usually the mother. The mothers, who were representative of the main study population of parents, were aged 30 years or more, just over half had previous children, most lived with a partner, approximately half were from Black and minority ethnic groups and almost all had been educated beyond 16 years of age. Examples of the abnormal findings included white matter changes, enlargement of ventricles, thinning of corpus callosum and cystic periventricular leukomalacia. Further details regarding the sample and the recordings have been reported elsewhere.^16^ Three-quarters of parents accepted the offer of a copy of the recording.

**Table 1:** Details of the 36 audio-recordings of clinician–parent discussions

Babies scanned	43 babies: 23 boys, 20 girls 30 singletons, 5 sets of twins, 1 set of triplets Born at 25^+2^–32^+6^ weeks' gestation, median 30^+1^ weeks’ gestation Corrected age at time of scan mean and median 2 weeks, 5 days
Scanning result	19 MRI, 17 US 38 normal, 5 abnormal (4 MRI, 1 ultrasound)
Parents present	18 recordings 1 parent present (17 mothers, 1 father) 17 recordings mother and father present 1 recording mother and grandmother present
Recording	6–49 min, mean 25 min, median 12 min 28/36 parents wished to have a copy of the audio-recording

An overarching theme of ‘the communication interface’ and three key themes were identified, each with several subthemes (table 2). Each subtheme is described separately and illustrated by examples of open text (CL denotes clinician, F father and M mother).

**Table 2:** Key themes and subthemes of the overarching theme, ‘the communication interface’

The communication interface	
Key themes	Subthemes
Giving and receiving information	Lengthy and complex explanations You don't need to know this Misunderstandings and muddles
Managing the conversation	Asks questions, no time to answer Use of rhetorical questions Closed questions, blocking and controlling
Getting it right	Tuning in to parents’ concerns Using humour Reassurance and relaxed chat Reaching an understanding

### Giving and receiving information

This key theme describes some of the consequences that occur when clinicians endeavour to give parents new, detailed and complicated information. Three subthemes were identified; ‘lengthy and complex explanations’, ‘you don't need to know this’ and ‘misunderstandings and muddles’. The use of lengthy and complicated explanations reflects the challenges experienced when introducing biological constructs and terminology while at the same time giving functional explanations in lay language. This was particularly notable when the anatomy and function of different areas of the brain were described. Considerable and often lengthy detail was given, with little opportunity for interaction. In many cases in presenting this sort of detail, clinicians spoke continuously with few interjections by parents. When parents did speak this was usually to say ‘yes’ or ‘no’. It appeared that the clinicians were not anticipating much of a response to the stream of information and were ready to move on quickly in the information-giving process:

CL: So basically, when we look at the head scan, we first look at the surface of the brain and that's folded, like a walnut. Then the centre of the brain is connected to that surface by what is called the white matter and that is a tissue that is vulnerable in preterm babies. The brain has two sides, the left and right side and the two sides are connected together by a bridge of fibres called the corpus callosum. Essentially, it just allows messages to flow from one side of the brain to the other side, so one side knows what the other side is doing. Within each side of the brain there are natural cavities called ventricles into which, there may be bleeding…. If there were any bleeds, the ventricles might increase in size. If the bleed was small or if there wasn't any they just carry on growing normally. And then finally, we look at the part of the brain called the cerebellum and that lies at the back of the neck. That's thought to be important in terms of balance as well as memory…so this is an image of his brain taken this way, ok? So his face is facing this way and that's his soft spot, up here. That's his skull bone, that's the left side and that's the right side, ok? So in the centre of the brain, are these round areas here and the surface of the brain is this white line that's going around edges here. And the centre is connected to that surface by the white matter, which is this white tissue here, ok? And then we also talked about the natural cavities which are called the ventricles…the left side is bigger than the right side. That is fine because none of us is completely symmetrical, ok? So that's a normal scan…. Any questions?

(9503/9511, twins, 31^+4^ weeks' gestation, US, normal)

When the results of the scan were ‘abnormal’, the situation was more complex. The clinician's explanations were longer and often included direct repetition of information or explanation in a variety of ways apparently aimed at ensuring parents understood what was being said. The discussion started with reference to the baby's gestational age and the low risk of babies born at that gestation having a problem. Clinicians continued by indicating that there was something of concern on the scan, saying that there was an increased chance of the child actually having a problem and referred to the possible longer-term consequences.

CL: So this is your baby's brain here. This is the brain in the middle. The white around the edge is fluid and we all have fluid around our brain and the white in the middle is fluid…. So this fluid is normal and that's fine. What I'm going to show you now, is the brain itself. We're going to look at that in a bit more detail. So if I start at the top of the head, we're now right at the top here. So I'm going to bring the scanner down and show you the brain. This is the top of the head, we're now coming a little bit lower so we're about here now, and this looks fine. Then as we come down a bit lower, you'll see that down here there's a little white patch. Can you see the white patch?

(2891, singleton, 32^+1^ weeks' gestation, MRI, abnormal)

The mother responded ‘yes’, and the clinician described again what had been observed and then expressed concern about the finding:

CL: We are a bit worried. Normally, children born at this age would have a very low chance of having problems when they grow up and you'd expect everything to be fine. But this does increase the chance of having problems when you grow up and those problems are likely to be problems with movement. It maybe that the legs are stiff or the arms are clumsy or something like that, and this is something that your doctors will need to watch very carefully, because it can be helped by treatment. You can't completely cure it but you can make life a lot easier for children who unfortunately have these problems…

 (2891, singleton, 32^+1^ weeks' gestation, MRI, abnormal)

The need to provide contextual information inevitably resulted in large segments of the discourse consisting of lengthy explanations in the course of which the clinicians appeared to be trying to be honest, clear and empathetic. At the same time, they tried to ensure that the parents understood what was being said.

During information-giving about the anatomy of the brain, the structures were labelled to orientate parents and to facilitate the discussion that followed. However, at times they gave additional or less relevant detail. Parents were sometimes presented with terminology about which the clinician then immediately said the parent did not need to ‘know’, or ‘remember’:

CL: And then we also look at the centre which is formed by the basal ganglia and the thalami. Don't worry about that.

(4316, singleton, 30^+4^ weeks' gestation, MRI, normal)

CL: So I'm sure you know that the brain, it has two sides, right and left and there are actually fibres connecting the two sides called the corpus callosum. There's no need to know that.

(9664, singleton, 32^+1^ weeks' gestation, US, normal)

While some parents may have already been familiar with the medical language used, there was recognition of the difficulties some may have faced when complex and unfamiliar terms were used. There was also potential for misunderstandings as parents attempted to understand and remember this new terminology when at the same time they were advised that they did not need to retain the information. Thus, there was some evidence of confusion at the interface, with the difficulties for both groups being evident in the exchanges which at times seemed rather circular. To clarify points, clinicians sometimes referred back to earlier points in the discussion. On some occasions, parents felt able to say that they did not understand and in these cases, they expressed the need for the clinician to provide clearer information:

F: Can that [the risk of cerebral palsy] change the other way? Can that improve?

CL: If she's got it?

F: No, you've said it's because of the thinning out, can that improve?

CL: No, it doesn't improve…remember I said the brain has two sides, right. The left and the right side and the two sides are connected together by these fibres, which form like a bridge that sends messages from one side of the brain to the other one. So it does it like on both sides, so the one side knows what the other side is doing. So that is thinned out as well. So it's not just the ventricles only. It's the ventricles plus this and then remember I said at the beginning that we're looking at the, the white matter is the tissue that is immediately vulnerable in preterm babies to having problems. So we're seeing some changes on that too which we think are to do with the brain trying to repair itself-

F: Yes, I don't understand.

M: You're not really explaining yourself.

F: We don't understand what you're. You're saying about, just the thinning by itself, what does that mean?

(1365, singleton, 29 weeks' gestation, MRI, abnormal)

In other situations, the clinicians recognised a need for clarification, although this did not always seem to work:

CL: And when you look at the picture, you'll see why. But what we're telling you is what is proven about the prognostic value of the scanner. So actually, it does give us more information at the moment-

M: But none of it might be useful.

CL: It may not…in terms of what it tells you about the future. It is more accurate, there's no doubt. We can see more, but what we've told you is based on what we've seen. So in that sense it is more information, it's much more information. Sorry, I'm going to rephrase that. It's many more facts, whether it's more knowledge is different.

(1622, singleton, 28^+2^ weeks' gestation, MRI, normal)

### Managing the conversation

The discussions were managed in a number of ways to ensure essential information was given. This approach generally centred on the use of questions. This included clinicians asking the parents questions but giving them no time to answer and the use of rhetorical or closed questions (requiring only ‘ok’, ‘yes’ or ‘no’ responses) to control the flow of information. There was very little evidence in the recordings of parents taking steps to manage the discussion and in their dependent role in this discussion this is not surprising.

In providing information clinicians often punctuated what they were saying with ‘ok?’ The intonation suggests that this was being posed as a question. However, this was generally followed by little or no pausing, and thus did not function as such. This may reflect the clinician's usual pattern of speech but in this context seemed to be a way of checking that the parents were still ‘with’ them and emphasising the points made. It could also have marked a change in direction or was a way of stressing that the results they were giving were satisfactory. The only response options parents seemed to have were to say ‘yes’ or ‘no’. However, it was very rare for parents to say ‘no’ at this point. As the clinician was clearly ready to move on, giving an opportunity for questions can seem disingenuous as parents were often given limited or no time to answer:

CL: So if you're born below 33 weeks you have a risk of about 9%, yes. That's what people have calculated the cerebral palsy risk, this is a risk, ok? So we're, there's a risk factor in this, being born preterm. And if you're born below 28 weeks then the risk is slightly higher at 14%, ok? But between 29 and 32 weeks, it's about 6%.

(6705, singleton, 25^+4^ weeks' gestation, MRI, normal)

Clinicians also asked rhetorical questions which can be an effective way of giving information. Familiarity with the type of questions parents often ask supports the use of this approach. Answering rhetorical questions can also be a useful way of supporting parents who for whatever reason feel unable to ask questions themselves. In the following example, the clinician continued with further contextual information about the risks for later problems:

CL: Now the nice thing about having the scans is that we can change that background risk by looking at the scan and saying how does that update our knowledge? Does that improve our understanding of what we're going to see? And in fact, the scans do that.

(2106/2131/2144, triplets, 28^+6^ weeks' gestation, US, normal)

Other ways in which clinicians managed the discussions were the use of closed questions, blocking parents' questions, redirecting the conversation and drawing the discussion to a close. These approaches appear to have been used to ensure the prognostic focus of the discussion was kept, building on the points previously covered. This seemed to involve shutting down other possible conversational pathways:

CL: So actually, we're very pleased with that and we can give you a lot more detail if you want it. But that's probably all we need to say isn't it?

(1784, singleton, 30^+4^ weeks' gestation, US, normal)

M: No, I just mean, like scans in general, like if you were to do one later, could you find something?

CL: Good question. Now let's talk about one thing at a time because we…

(6718, singleton, 27^+2^ weeks' gestation, US, normal)

In some cases, parents asked questions that the clinician appeared to not want to answer at all. This seemed to be because the clinician felt the depth of explanation required would not be helpful to the parent. In the following example having previously been told that his baby's scan was normal, the father asked what would be the implications for a baby of having a ventricular bleed:

F: So what is the impact of that?

CL: Of the big bleed?

F: On the health of the baby?

CL: I'm not going to tell you, because it doesn't affect you. Honestly, because I'm going to start confusing you.

F: Ok, ok.

The discussion continued and towards the end, the clinician asked:

CL: Do you still want me to answer that other question?

F: No. I don't think I want to know now.

CL: Ok. That's why I didn't want to answer it.

F: It's not going to help me.

CL: Absolutely.

(6125, singleton, 31+5 weeks' gestation, MRI, normal)

### Getting it right

There were numerous examples throughout the discussions of the ways in which the clinicians facilitated the communication process. While there were few examples of topics initiated by parents, they responded enthusiastically and promptly to the approaches adopted by the clinicians. These approaches included tuning in to parents' concerns, using humour, providing reassurance and chatting and reaching an understanding.

The clinicians were aware that the key things that the parents wanted to know were ‘Is my baby ok?’ or ‘Is my baby normal?’ The anxiety of some of the parents was evident to the clinicians, and they reassured parents at the earliest opportunity. This was much more straightforward when the scans were ‘normal’. The clinicians were open about saying so, the language they used was simpler, the statements were shorter, they emphasised that the scan was normal throughout the discussion and used the scan pictures to confirm that nothing of concern had been identified.

CL: I can see you're getting worried so I'm going to tell you now the scans are normal.

M: Oh ok, yes ((laughs))…. Yes, that's good to know, yes.

CL: I think it's important that we tell you everything about the scans, but I could just see you getting worried.

(2106/2131/2144, triplets, 28^+6^ weeks' gestation, US, normal)

CL: Look at the picture here, it looks quite nice. It's quite proportionately normal and it's quite symmetrical, which is also a very good thing to have.

(7519, singleton, 26^+2^ weeks' gestation, MRI, normal)

It was more challenging when the scan indicated a more mixed or uncertain situation, as was shown for example with earlier quotations, reflecting more muddled communication between clinicians and parents.

During the discussions, the clinicians often made positive comments which seemed to be a way of reassuring the parents and normalising what was seen on the scan. As might be anticipated, humour was not used in the discussions when abnormal results were given. However, humour did sometimes feature when normal results were discussed, commonly lightening up of the discussions. Parents seemed to appreciate this and usually responded by laughing or ‘playing along’ with the joke. Conversely, a few parents introduced humour, to which the clinicians responded:

CL: Down the bottom of her head now, here's her teeth. You may not think she's got teeth but-

M: Ok ((laughs)).

CL: Those are her teeth.

M: Right.

CL: Does she need orthodontic work? I don't know.

M: ((laughs)).

(5175, singleton, 27^+6^ weeks' gestation, MRI, normal)

F: They've got slightly different shaped brains.

CL: Yes.

M: Of course the female brain is far more superior. You know that, don't you? ((all laugh)). It's with all the multi-tasking.

CL: Yes, that's true ((laughs)). (9569/9576, twins, 30^+5^ weeks' gestation, MRI, normal)

Clinicians could also engage in more general conversation with the parents about their baby's time on the neonatal unit, how things have been since discharge home and their experience as parents as well as their day at the scanning appointment. This relaxed chat took place at the beginning or end of the information-giving. It was non-technical, and it enabled parents and clinicians to behave in a more conventionally equitable way in the social interchange:

CL: Well they've obviously done very well. You must have had a very scary time.

M: When they were born, at 29, 28 weeks, 6 days, so 29 weeks practically, but I was very fortunate that I'd gone in to X ((hospital))…and on the Wednesday they managed to give me the steroid injection.

CL: Yes.

M: Which, I didn't think I was in labour, I was like oh, right, ok and then-

CL: And they got there in time.

M: Yes. So I had two, three days of that, so that was fantastic. And then when I went to the unit, I met other mothers and their babies were 23, 24 weeks and they had been through the hell you know-

CL: Yes.

(2106/2131/2144, triplets, 28^+6^ weeks' gestation, US, normal)

When what was seen on the scan was of concern, reassurance was also used:

CL: Ok, well when they see her they will be looking, you know, can she move her legs, those kind of things, following her milestones. And we do that because we don't want to miss anything so we can do something about it early…. Don't worry too much about it. I'm telling you because you need to know, but I'm not telling you because I think that's exactly what's going to happen. It's just a chance. Alright?

(4986, singleton, 32^+6^ weeks' gestation, MRI, abnormal)

Parents would commonly take time and in reaching an understanding would engage more in the discussion. In the active process of communication, they used a range of strategies in trying to seek information, aid recall and demonstrate their understanding by using the terminology they had acquired, repeating or summarising, confirming understanding, completing the clinician's sentences and asking questions.

CL: So if a baby's…, had a normal scan at this time, then we expect them to be able to have, to walk, jump, run, talk, do all of those things-

F: Right.

CL: -on time as usual. However, the risk with attention, memory, concentration and difficulties at school-

F: Stays the same.

CL: -stays the same.

(1435, singleton 29^+6^ weeks' gestation, US, normal)

Repetition was particularly salient in some interactions and seemed to reflect working towards a shared understanding. There was sometimes matching by the clinician as well as the parents with phrases, words and parts of phrases being repeated:

M: Yes, this one is white dots.

CL: Yes, that's right. Where there are white dots, absolutely right.

(2891, singleton, 32^+1^ weeks' gestation, MRI, abnormal)

CL: Are you alright? ((long pause)) remember that we talked about a risk. Remember we're not talking about something that's definite. Ok? So we're not saying this is definitely what's going to happen. We're just saying it's a chance.

F: It's a chance ((whispered)).

(4986, singleton, 32^+6^ weeks' gestation, MRI, abnormal)

Parents confirmed their understanding in different ways. The echoing use of ‘just’ by parent and clinician in the following example illustrates a shared summarising of the information and shows how a parent had reframed the risk, emphasising the limits of prediction from the images they had been shown:

CL: At this stage it's just knowledge, that this is what she has.

F: So it's just like the ventricles.

CL: Yes.

F: It's just observation.

CL: Yes, yes.

M: Ok, alright.

(1365, singleton, 29 weeks' gestation, MRI, abnormal)

Parents also confirmed their understanding of the results of the scans by summarising the key points:

CL: So as far as I know, there was no evidence today of any problems on the scans.

M: Yes.

F: So in other words, it's like any prognosis, there's no certainty that everything will be fine, but there's no symptoms to indicate that you're worried about anything.

M: Well exactly, I mean it's just like any other child whose born preterm. It's like you know, it's only if something develops that you-

F: There's no other risk factor other than they were born preterm.

(2047/2059, twins, 28^+6^ weeks' gestation, MRI, normal)

## Discussion

Key topics were covered in all the audio-recorded discussions with parents (box 1) across the 3 years of the study. The main themes and subthemes described here reflect the communication process, the way this was managed, the needs and goals of the participants and some mismatches that occurred. The analysis illustrates the challenges that clinicians face during such discussions. This was particularly the case when abnormal scan results had been identified, and complex messages about an uncertain future had to be given and received. The general content of the discussions was a function of the scanning process and the findings. The communication interface was largely managed by the clinicians.^14^ Their knowledge and experience put them in a powerful role in this interaction with parents who are often aware of the imbalance and their dependence on the medical staff.^6^ The clinicians had control over the flow of information, a position which contrasted markedly with that of the parents of the preterm infants. This has been identified in others studies of information-giving in neonatal care.^8^ ^23^ While this inequity is inevitable, the clinicians appeared to be aware and made efforts to moderate the imbalance by repeating and summarising information and taking a lighter approach to aspects of the discussion when this was appropriate.

In some instances, the parents’ prior knowledge and level of understanding and the questions they asked could have diverted the discussion. On these occasions, clinicians counterbalanced providing essential information with at the same time being responsive and empathetic to the parents’ needs. As in other qualitative studies,^14^ ^15^ the analysis which was facilitated by the specifically developed framework^16^ highlighted a number of issues. Relatively short discussions involving lengthy descriptions and explanations using unfamiliar terminology allow little time for parents to respond or explore issues of concern. Signposting, longer pauses and the clinician's use of open questions appeared to facilitate parental understanding which in turn may have enabled them to more readily reflect on and respond to the information they were given. Other strategies such as ‘relaxed chat’ and the use of humour have also been found to reduce parental anxiety.^6^

The use of complex terminology to describe features of the scanned images can be problematic. There is an argument for the use of correct use of terms: some parents may already be accustomed to them and for some families the language will become all too familiar in the future. Nevertheless, if a parent's first encounter with such terminology is an occasion when they are also being given an indication of their child's prognosis, the unfamiliar language may provoke further anxiety. Telling the parents that ‘you don't need to know this’ or ‘you don't have to remember this’ may have been the clinician's way of focusing parents on the essential information. However, this approach may seem rather dismissive and begs the question; if the parents do not need to know, why tell them in the first place?

It is important to be aware that the findings are based on discussions that were part of a research study, including a trial of information-giving based on MRI or US rather than routine clinical practice and interactions that occur outside this context may therefore differ. Nevertheless, as in many healthcare contexts there was relatively little time for parents to formulate questions and discuss their concerns. We would argue that the ways in which the participants interacted are unlikely to differ substantially. Clinicians have limited time and are usually talking from a basis of knowledge and experience, and parents are generally in the position of being less well informed, usually with limited experience of neonatal care and having a preterm baby. We were unable to explore the impact of clinician experience and training on the interactions recorded, however, both factors are likely to contribute to variation in practice. As neonatal specialists with experience in giving diagnostic and prognostic information, the way in which they did so may have differed from that of developmental neurologists, however, no similar studies were available with that clinician group.

The fact that 80% of ‘family units’ involved in the main study consented to their discussion with the clinician being audio recorded suggests that most parents would be comfortable with this approach, as in other research contexts.^4^ ^11^ ^13^ Using MRI images to talk over prognosis may become more common, particularly when there is concern about possible adverse outcomes. It is important also to understand the parents' perspective of these discussions and how they made sense of the information they were given about possible future of their young babies.^10^ The meaning that parents take away from these discussion about their baby's future will be further explored in parents' responses to questionnaires and qualitative interview data collected at one and two years after these clinician–parent discussions.

## Conclusions

The communication interface appears to be a rather uneven one in which the emphasis is on what the clinician sees and feels a responsibility to explain. The language and constructs used in the discussions reflect a complex situation in which there may be a compromise between the needs of individual parents and the information-giving required. This study highlights the importance of making time to talk to parents and understanding their perspective and level of knowledge. The need to revisit specific issues or points within a discussion, especially when the findings are mixed or of concern, has been established.

We were in a privileged position in being able to analyse such recordings and to explore information-giving and receiving. Being able to do so in a clinical context is uncommon. The insights gained have the potential to inform practice in talking to parents of preterm and sick infants and in training and supporting clinicians and other health professionals in working with parents.
